# Lymphopenia during chemoradiation—foe or friend

**DOI:** 10.3332/ecancer.2020.1109

**Published:** 2020-09-24

**Authors:** Vijay M Patil, Gunjesh Kumar Singh, Vanita Noronha, Amit Joshi, Nandini Menon, Sarbani Ghosh Lashkar, Vijayalakshmi Mathrudev, Kavita Nawale Satam, Sadaf Abdulazeez Mukadam, Kumar Prabhash

**Affiliations:** 1 Department of Medical Oncology, Tata Memorial Hospital, Mumbai 400012, India; 2 Department of Radiotherapy, Tata Memorial Hospital, Mumbai 400012, India; # Co-first author

**Keywords:** lymphopenia, head-and-neck cancer, chemoradiation

## Abstract

**Background:**

Severe lymphopenia during treatment is considered to be a poor prognostic factor. The current literature lacks information regarding its impact on various outcomes in locally advanced head-and-neck cancer patients in a prospective setting.

**Methods:**

We recently published a randomised study comparing cisplatin–radiation with nimotuzumab cisplatin–radiation. The database of this study was used for the present analysis. The impact of severe lymphopenia (grade 4 lymphopenia) on progression-free survival (PFS), locoregional control (LRC) and overall survival (OS) was studied using the Kaplan–Meier method and Cox regression analysis. The binary logistic regression analysis was used to see the effect of various factors on the development of severe lymphopenia.

**Results:**

We had a total of 536 patients, of which 521 patients (97.7%) developed lymphopenia. Grade 1 lymphopenia was noted in 10 (1.9%) patients, grade 2 in 100 (18.8%), grade 3 in 338 (63.1%) and grade 4 in 73 (13.7%) patients. The median PFS was 20.53 and 60.33 months in severe and non-severe lymphopenia, respectively (hazard ratio, 0.797; *p*-value = 0.208). The median duration of LRC was 56.3 months in severe lymphopenia, whereas it was not reached in non-severe lymphopenia (hazard ratio, 0.81; *p*-value = 0.337). The median OS was 28.46 versus 47.13 months in severe and non-severe lymphopenia, respectively (hazard ratio, 0.76; *p*-value = 0.11). Of various risk factors, gender was significantly associated with severe lymphopenia.

**Conclusion:**

The occurrence of severe lymphopenia was not significantly associated with the outcomes. Gender is the only risk factor significantly linked to severe lymphopenia.

## Introduction

Lymphocytes play a key role in tumour response; however, they are also the most sensitive cells to chemoradiation [1]. According to Campian *et al* [2], lymphopenia is commonly seen during the administration of chemoradiation to head-and-neck cancer (HNC) patients. In a prospective study, combined treatment with cisplatin and radiotherapy induced lymphopenia in 78% of patients [3]. In another phase I trial, lymphopenia (grade ≥ 3) was seen in 90% of patients receiving nimotuzumab along with chemoradiation for oesophageal cancer [4]. It has been postulated that lymphopenia hampers the body’s ability to destroy tumour cells [5].

HNC shows the presence of many infiltrating immune cells including lymphocytes—hence, it is called an immunogenic tumour [6]. Furthermore, the pre-treatment lymphocyte counts can be of prognostic value in HNC as proposed by some previous studies [7]. Reportedly, low absolute numbers of CD3+, CD4+ and CD8+ T cells have been found in patients with head-and-neck squamous cell carcinoma in comparison to the normal controls. In addition, patients with active disease tend to show significantly lower CD3+ and CD4+ T cell counts than those with no disease [2].

The occurrence of lymphopenia and its impact on outcomes have never been evaluated in any prospective study in HNC patients. In view of this lacuna in the current literature, we performed this *post hoc* analysis to study the impact of severe lymphopenia on outcome (progression-free survival, loco-regional control and overall survival) in HNC patients undergoing radical chemoradiation.

## Materials and methods

**Data set**—We have recently published the results, outcome and adverse effect of a phase III randomised trial. In this study, 536 patients were randomised 1:1 to receive either radical radiotherapy (66–70 greys) with concurrent weekly cisplatin (30 mg/m^2^) (CRT) or the same schedule of CRT with weekly nimotuzumab (200 mg) (NCRT). The primary endpoint was progression-free survival (PFS); key secondary endpoints were the duration of loco-regional control (LRC) and overall survival (OS) [8]. The database of this study was used for the present *post hoc* analysis.

**Data collection**—From the above-mentioned data set, the data of lymphopenia in addition to demography and outcome were extracted, and the following were noted:

- The occurrence of lymphopenia from week 0 to week 6 of treatment was taken into account.
- The maximum grade of lymphopenia as per the CTCAE criteria 4.03.
- Absolute lymphocyte counts during chemoradiation.

### Definition of lymphopenia as per the CTCAE criteria 4.03

- Grade 1 lymphopenia: < lower limit of normal—800/μL
- Grade 2 lymphopenia: 500–800/μL
- Grade 3 lymphopenia: 200–500/μL
- Grade 4 lymphopenia: <200/μL

Grade 4 lymphopenia was considered to be severe lymphopenia, whereas others (grade 1–3) were considered as non-severe lymphopenia.

**Endpoints (outcomes)**—To ascertain the impact of lymphopenia, the following endpoints were considered:

- PFS was defined as duration from the date of randomisation to the first evidence of tumour progression.
- LRC was defined as the time between the date of randomisation and the date of local or regional relapse.
- OS was calculated as the time from the date of randomisation to the date of death from any cause.

### Statistics

Statistical analysis was performed using the SPSS version 20 and R version 3.5.3. A descriptive analysis was performed. The continuous variables were expressed in median value along with its range, whereas the non-continuous/ordinal variables were expressed in percentage (%) along with 95% confidence interval (CI). The impact of severe lymphopenia on PFS, LRC and OS was studied using the Kaplan–Meier method and Cox regression analysis. The binary logistic regression analysis was used to see the impact of factors (age, gender, Eastern Cooperative Oncology Group performance score (ECOG PS) and chemotherapy regimen) on the development of severe lymphopenia. A *p*-value of 0.05 was considered to be significant.

## Results

### Baseline characteristics

The detailed baseline characteristics of these patients have already been published [8]. The median age was 54 and 55 years in the CRT and NCRT arm, respectively. In the CRT arm, there were 86.2% of males and 13.8% of females, whereas, in NCRT arm, there were 84.3% of males and 15.7% of females. Stage IVA (64.2% versus 66%) was the most common stage, followed by stage III (32.5% versus 29.9%) and stage IVB (3.4% versus 4.1%) in CRT and NCRT, respectively. In both the arms, 99.6% of patients had ECOG PS of grade 0–1, whereas 0.4 % had grade 2. The baseline characteristics and treatment details of the patients are shown in Table 1.

### Lymphopenia

**Figure 1.**
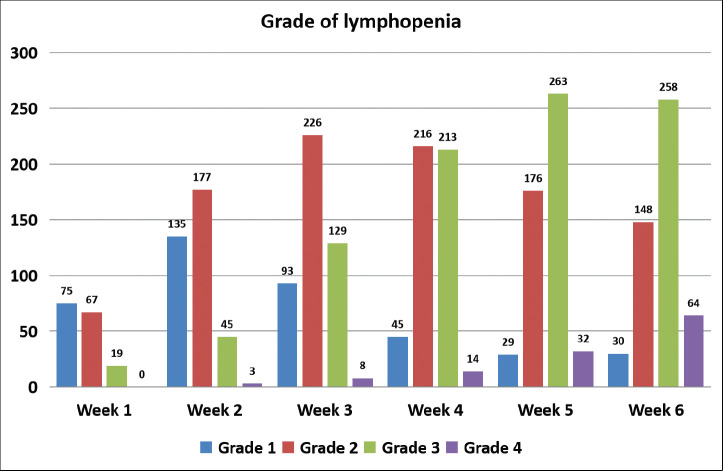
Week-wise incidence and grading of lymphopenia.

The acute onset of lymphopenia was captured in 532 of these 536 patients. Of 532 patients, 521 patients (97.7%) developed lymphopenia. Grade 1 lymphopenia was noted in 10 (1.9%) patients, grade 2 in 100 (18.8%), grade 3 in 338 (63.5%) and grade 4 in 73 (13.7%) patients. Week-wise incidence and grade of lymphopenia are shown in Figure 1.

### Factors impacting lymphopenia

Amongst various factors, only gender was found to be significantly associated with grade 4 lymphopenia (Table 2). Severe lymphopenia was seen in 12.6% of males (56 out of 454) and 22.4% of females (17 out of 76) (odds ratio: 2.043; 95% CI: 1.108–3.766; *p*-value = 0.022).

### Impact on outcome

**Figure 2.**
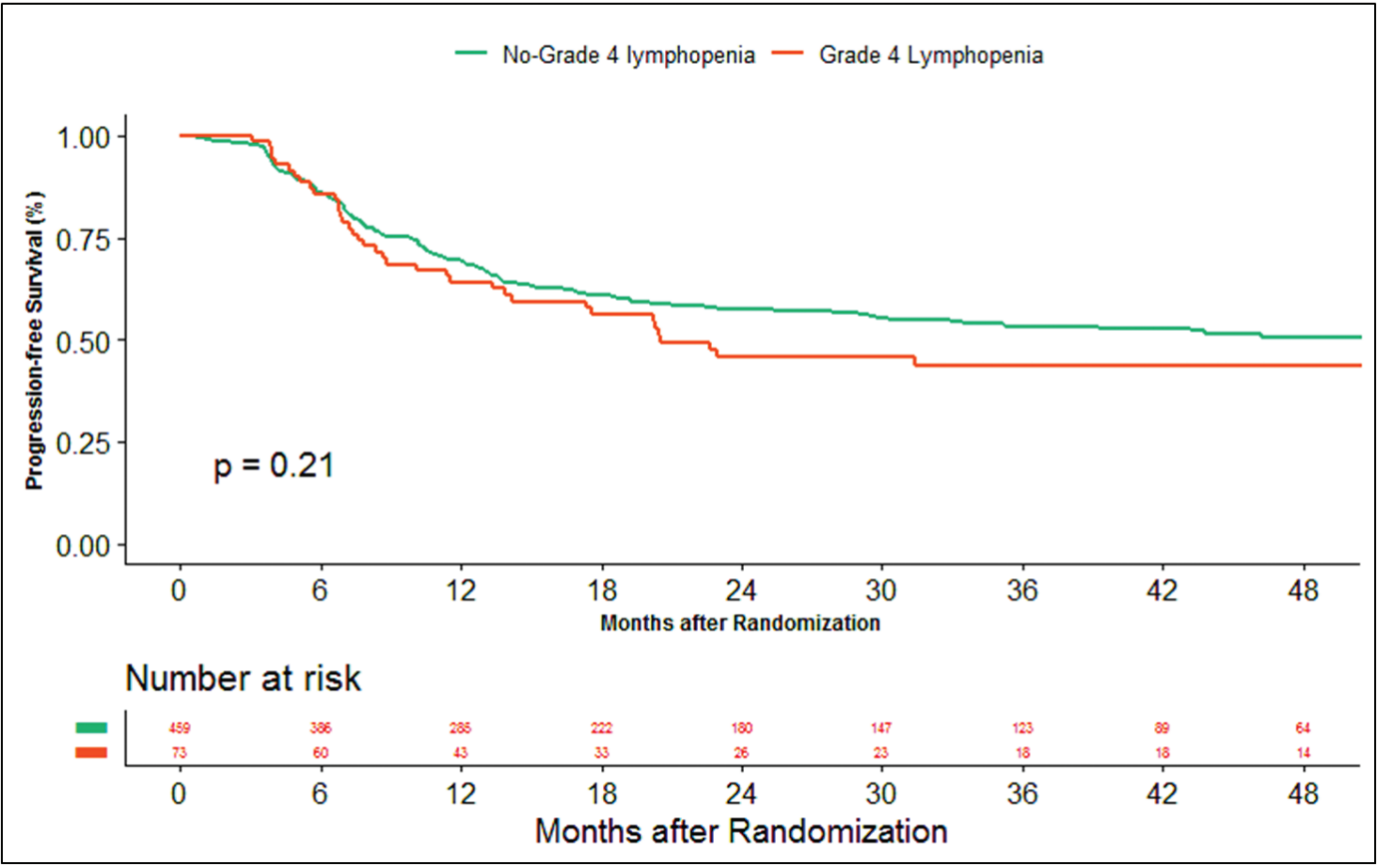
Kaplan–Meier curve showing progression-free survival in grade 4 (severe) and non-grade 4 lymphopenia (non-severe).

**Figure 3.**
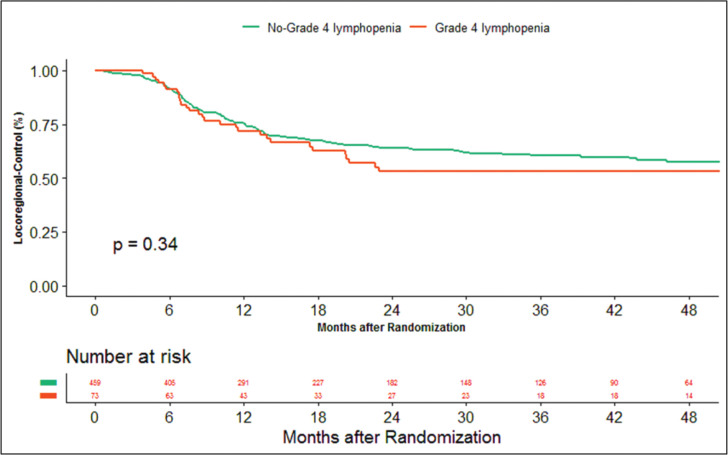
Kaplan–Meier curve showing loco-regional control in grade 4 (severe) and non-grade 4 lymphopenia (non-severe).

**Figure 4.**
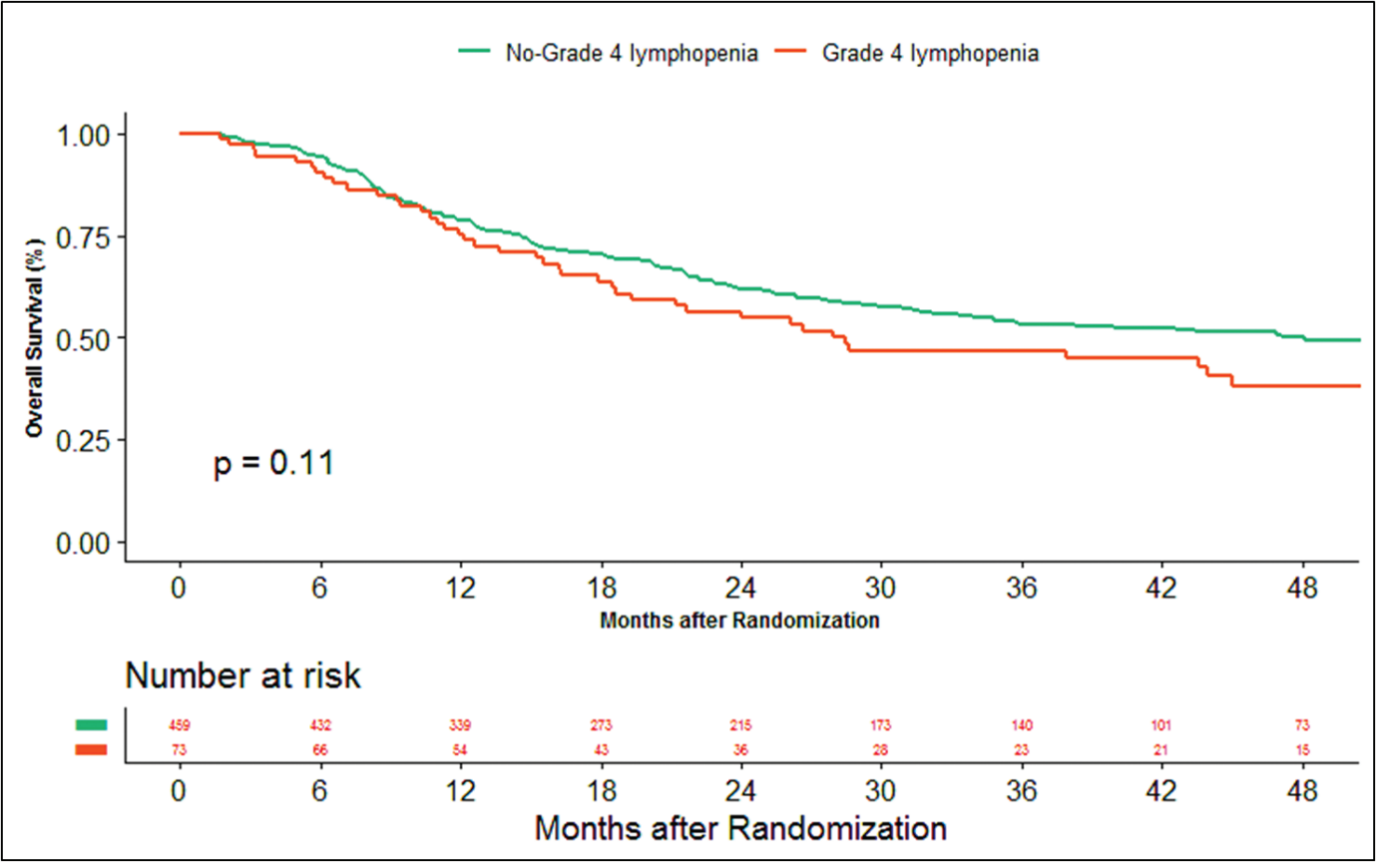
Kaplan–Meier curve showing overall survival in grade 4 (severe) and non-grade 4 lymphopenia (non-severe).

The median duration of follow‐up of this study was 39.13 months. At the time of censoring, 38 (*n* = 73) and 196 (*n* = 459) patients experienced disease progression with severe and non-severe lymphopenia, respectively, and their median PFS was 20.53 (95% CI: 6.87–34.18) and 60.33 months (95% CI: NA), respectively (hazard ratio: 0.797; 95% CI: 0.56–1.12; *p*-value = 0.208) (Figure 2). Loco-regional failure was observed in 30 patients with severe lymphopenia (*n* = 73), whereas it was seen in 160 (*n* = 459) in those with non-severe lymphopenia. The median duration of LRC was 56.3 months (95% CI: 0–115.78) in severe lymphopenia, whereas it was not reached in non-severe lymphopenia (hazard ratio: 0.81; 95% CI: 0.55–1.21; *p*-value = 0.337) (Figure 3). There were 42 (*n* = 73) versus 197 (*n* = 459) deaths, and the median OS was 28.46 (95% CI: 11.64–45.28) versus 47.13 (95% CI: NA) months in severe and non-severe lymphopenia, respectively (hazard ratio: 0.76; 95% CI: 0.54–1.06; *p*-value = 0.11) (Figure 4).

**Supplementary Figure 1.**
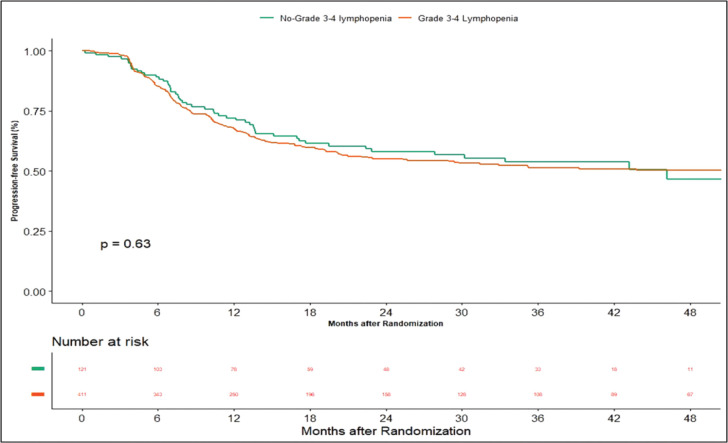
Kaplan–Meier curve showing progression-free survival in grade 3–4 and non-grade 3–4 lymphopenia.

**Supplementary Figure 2.**
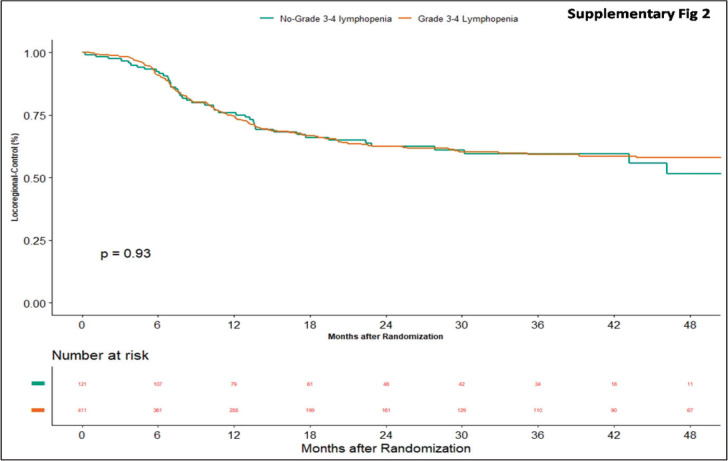
Kaplan–Meier curve showing loco-regional control in grade 3–4 and non-grade 3–4 lymphopenia.

**Supplementary Figure 3.**
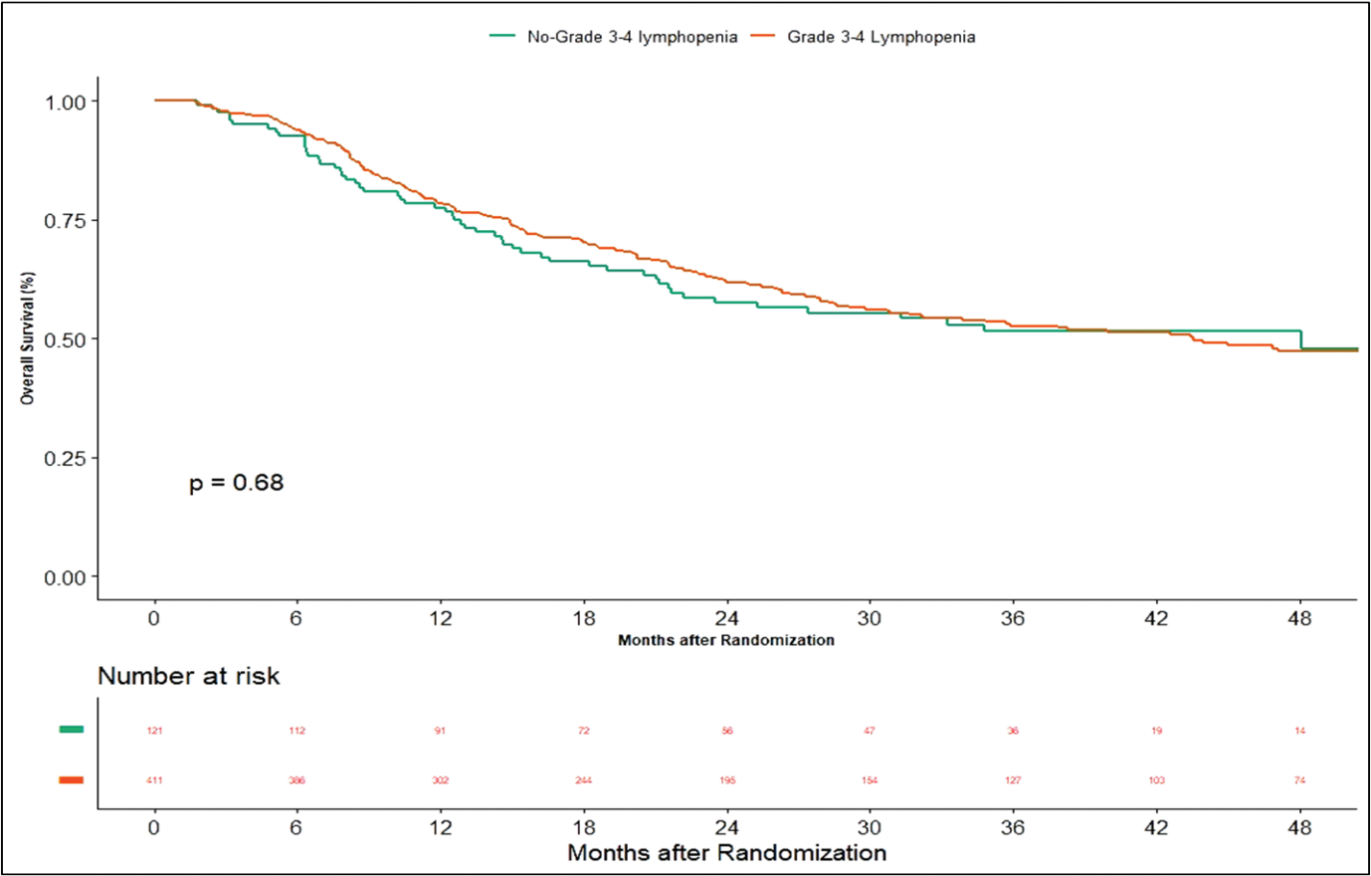
Kaplan–Meier curve showing overall survival in grade 3–4 and non-grade 3–4 lymphopenia.

Apart from this, we also did an additional analysis to see any impact of lymphopenia on the outcomes. Here, we compared combined grade 0–2 lymphopenia with combined grade 3–4 lymphopenia. Of 532 patients, 121 patients (22.74%) developed lymphopenia of grade 0–2, whereas grade 3-4 lymphopenia was seen in 411 (77.25%) patients. At the time of censoring, 51 (*n* = 121) patients with grade 0–2 and 183 (*n* = 411) patients with grade 3–4 lymphopenia experienced disease progression. The median PFS of grade 0–2 and grade 3–4 lymphopenia was 46.2 (95% CI: 22.4–NA) and 56.3 months (95% CI: 22.9–NA), respectively (*p*-value = 0.63) (Supplementary Figure 1). Loco-regional failure was observed in 44 patients with grade 0–2 lymphopenia (*n* = 121), whereas it was seen in 146 (*n* = 411) patients with grade 3–4 lymphopenia. The median duration of LRC did not reach in both grade 0–2 and grade 3–4 lymphopenias (*p*-value = 0.93) (Supplementary Figure 2). There were 56 (*n* = 121) versus 183 (*n* = 411) deaths, and the median OS was 48.1 (95% CI: 21.7–NA) versus 43.5 (95% CI: 31.6–60.6) months in grade 0–2 and grade 3–4 lymphopenia, respectively (*p*-value = 0.68) (Supplementary Figure 3).

## Discussion

The existence of peripheral immune alterations in cancer patients was shown for the first time in the mid-1970s by Bone and Lauder [9]. Lymphopenia can be functional or treatment induced. Functional lymphopenia has been reported in advanced or metastatic breast cancer, colon cancer and hepatocellular carcinoma [7]. It is associated with dismal outcome. Furthermore, in various malignancies such as carcinoma breast, carcinoma ovary, non-Hodgkin lymphoma, diffuse large B-cell lymphoma, T-cell lymphomas, sarcomas and colon cancer, lymphopenia detected before treatment and early after starting chemotherapy (5–15 days) or radiotherapy is associated with a worse PFS and OS [7]. Its impact on relapse-free survival (RFS) and OS in various malignancies is shown in Table 3 [10–15]. In addition, the severity of lymphopenia has been reported as an independent prognostic factor of PFS and OS. According to Manuel *et al* [16], severe lymphopenia (<200/mm^3^) in metastatic breast carcinoma has a strong impact on patient survival and also reported 40% and 90% reduction of the median OS or PFS, respectively. Head-and-neck squamous cell carcinoma (HNSCC) is known as an immunogenic tumour showing the presence of infiltrating lymphocytes and other immune cells [17]. The previous studies have suggested the important role of pre-treatment lymphocyte counts with regard to their association with prognosis in HNSCC [7]. However, Campian *et al* [2] in a retrospective study found no significant difference in PFS and OS between patients with TLC < 500 cells/mm^3^ and those with TLC ≥ 500 cells/mm^3^. Similarly, in this study, there was no statistically significant impact of severe lymphopenia on various outcomes (PFS, LRC and OS).

Various studies have considered grade 3–4 as severe lymphopenia, and it was reported in 61% of HNC patients and 53% of cervical cancer patients by Campian *et al* [2] and Wu *et al* [18], respectively [18], whereas Zhou *et al* [17] found grade 4 lymphopenia in 31% of oesophageal cancer patients during chemoradiation. In this study, we found lymphopenia in 97.7% of patients, where, in 13.7% of patients, it was grade 4.

Older age, female gender, accelerated radiotherapy and certain chemotherapy agents (cisplatin, cyclophosphamide, methotrexate and taxanes) have been reported as significant risk factors for the development of lymphopenia [7, 19]. In this analysis, we analysed these factors. Of age, gender, ECOG performance score and chemotherapy regimen (cisplatin versus cisplatin + nimotuzumab), gender was the only significantly associated factor with severe lymphopenia.

This first prospective study to enlighten lymphopenia and its impact on survival outcomes in head-and-neck cancer patients is unique. There are several limitations to this study. First of all, lymphopenia occurring only during treatment was analysed, so the post-therapy lymphopenia status cannot be commented on, although it is unlikely to witness post-treatment lymphopenia in a weekly chemotherapy schedule. Second, these data are applicable only for weekly cisplatin-based chemoradiation as there are different haematological toxicities in once a week versus once a 3-week cisplatin schedule [20].

## Conclusion

Lymphopenia is fairly common in HNC patients during chemoradiation. Gender is an important factor associated with severe lymphopenia; however, there is no significant impact of severe lymphopenia on PFS, LRC and OS.

## Conflicts of interest

None declared.

## Declaration of funding

None.

## Figures and Tables

**Table 1.** Baseline characteristics and treatment details.

Characteristics	Grade 0–3 lymphopenia	Grade 4 lymphopenia	*p*-value
Age ≥60 years <60 years	135 (29.4%) 324 (70.6%)	23 (31.5%) 50 (68.5%)	0.783
Primary site Oral cavity Oropharynx Larynx Hypopharynx	2 (0.4%) 232 (50.5%) 136 (29.6%) 89 (19.4%)	1 (1.4%) 35 (47.9%) 18 (24.7%) 19 (26%)	0.286
Oral tobacco Yes No	203 (44.2%) 256 (55.8%)	36 (49.3%) 37 (50.7%)	0.448
Tobacco smoking (bidi) Yes No	239 (47.9%) 220 (52.1%)	32 (43.8%) 41 (56.2%)	0.209
Tobacco smoking (cigarette) Yes No	91 (19.8%) 368 (80.2%)	10 (13.7%) 63 (86.3%)	0.261
HPV (p16) * Positive Negative Equivocal Not done	22 (9.5%) 159 (68.5%) 0 51 (20.7%)	1 (2.9%) 28 (80%) 1 (2.9%) 5 (14.3%)	0.067
Cisplatin cumulative dose—200 mg/m^2^ Yes No	365 (79.5%) 94 (20.5%)	52 (71.2%) 21 (28.8%)	0.126
Nimotuzumab received Yes No	247 (53.81%) 212 (46.2%)	34 (46.57) 39 (53.43%)	0.191
Total radiotherapy dose ≥70 Grey <70 Grey	403 (87.79%) 56 (12.20%)	57 (78.08%) 16 (21.91%)	0.873
Radiotherapy received (100% of planned dose) Yes No	431 (93.9%) 28 (6.1%)	68 (93.2%) 5 (6.2%)	0.794
Radiotherapy received (100% of planned dose) Yes No	432 (94.1%) 27 (5.9%)	69 (94.5%) 4 (5.5%)	1.00

* HPV status was detected by p16 immunohistochemistry staining and is reported according to the College of American Pathologists criteria for patients with oropharyngeal cancer. Samples for which testing was possible in patients with oropharyngeal cancer.

**Table 2.** Factors and their impact on severe lymphopenia

Factors impacting lymphopenia	*p*-value	Odds ratio (OR)	95% Confidence interval (CI)
Age	0.545	0.846	0.493–1.453
Gender	0.022	2.043	1.108–3.766
ECOG PS	0.793	0.921	0.499–1.77
Regimen (CRT versus NCRT)	0.360	1.263	0.767–2.080

**Table 3.** Impact of lymphopenia on relapse-free survival (RFS) and overall survival (OS).

S.no.	References	Disease	Lymphopenia	Impact on RFS (*p*-value)	Impact on OS (*p*-value)
1	Ray-Coquard *et al* [10]	Sarcoma	GL	NE	0.05
2	Saroha *et al* [11]	Renal cell carcinoma	GL	NE	0.012
3	Ceze *et al* [12]	Colon cancer	GL	0.048	0.003
4	De Giorgi *et al* [13]	Breast cancer	GL	0.001	0.001
5	Ray-Coquard *et al* [10]	Non-Hodgkin lymphoma	GL	0.002	0.04
6	Tredan *et al* [14]	Breast cancer first relapse	CD4+ lymphopenia	NE	0.00001
7	Tredan *et al* [14]	Breast cancer > second relapse	CD4+ lymphopenia	0.183	0.086
8	De Angulo *et al* [15]	Ewing sarcoma	GL	NE	0.007
9	Indexed data	Head-and-neck cancer	GL	NE	0.11

RS = Relapse-free survival;

OS = Overall survival;

GL = Global lymphopenia;

NE = Not evaluated.
